# Event and Comment

**Published:** 1921-05

**Authors:** 


### DENTAL REGISTER

THE MAGAZINE OF DENTISTRY

Vol. LXXV MAY, 1921 No. 5

### EVENT AND COMMENT

**Dangers from Oral Infections** We have heard so much about the dangers to which we are subjected from the innumerable accidents to injured and diseased teeth that we sometimes wonder if we are not too frequently scared more than we are hurt, and yet when we think of the terrible conditions we encounter in our every-day practices, and in our college clinics, we are almost ready to say that there is no such thing as safety in any oral conditions. If we think of the great chances for disease inception from oral sepsis in the average mouth, and compare it with the fact that when conditions are favorable the tremendous and serious results which may occur from slight wounds or more unsanitary conditions, we wonder how any one is saved or keeps well. We think there must certainly be a kind and merciful Providence which keeps us from our own selves and lives. The only help for us seems to be that we use our best scientific endeavors to obtain the best possible degree of general health that is possible, and then by careful scrutiny we secure the greatest precautions in prophylaxis and therapeutic activity, and so ward off impending dangers. Even then there will be enough menacing diseases that have intruded the body tissues insidiously without creating noticeable warnings to overcome our most active endeavors to evade them. We have thought we had rounded up the most insidious and persistent forms of microbic pestilences by our medications and surgical skill, and we were in a fair way to clean house; but now comes our bacteriological discoverers who tell us we have only begun to weed out the more persistent and active forms, and there are innumerable other "fleas to bite;" the big ones we have long known. It seems now our old microbes have learned to change their forms and they still sting as venomously as any others. With their changed forms and colors they even are more vicious than ever.

The game is becoming so complex and intricate that it seems as though we shall be compelled to adopt the most radical means we can devise, short of absolute destruction. Fire that is sufficiently fierce to kill we can't use, no more than we can administer poisons that endanger human life. There seems to be no logical method of curing infectious diseases except by the use of the extremes in prophylaxis and medical surgery.

In this emergency is it not worth while that we make a real effort to inaugurate a scheme of absolute preventive dentistry? Of course that is impracticable, we may think, and should only be thought of as a dream. But dreams have materialized, and some dreamers have been prophets. In fact, it does not seem impracticable that in the near future we may be paving the way for a new era in dental health and happiness.

We have failed to prevent dental caries by the operative and surgical measures, and only our crutches, in the way of dentures, are left as a last support. It is now time to begin real preventive efforts, starting with our two-year-olds, as an only hope.

